# Habitat preferences and conservation threats to Black-necked Cranes wintering in Bhutan

**DOI:** 10.1186/s40064-016-1923-0

**Published:** 2016-02-29

**Authors:** Rinchen Namgay, Sangay Wangchuk

**Affiliations:** Department of Sustainable Forestry, Ugyen Wangchuck Institute for Conservation and Environment, Bumthang, Bhutan

**Keywords:** Black-necked Crane, Habitat, Conservation threats, Roosting, Bumthang

## Abstract

Black-necked Crane (*Grus nigricollis*) is a vulnerable Red list species whose populations are declining. However, little is known about Black-necked Cranes’ habitat requirements or the causes of their population decline. We identified Black-necked Cranes’ winter roost and foraging preferences of Black-necked Cranes in Bhutan during the winter of 2013–2014. Black-necked Cranes’ roosts were recorded using Garmin GPSmap 60CSx, while foraging preferences and threats to the birds were identified based on a survey of household heads (n = 107) residing within a 3 km radius of roost sites. We grouped the threats identified by the communities into four major categories, viz. biological, social, political and natural threats based on the relevance. Of the four major threats, communities residing within the roosting and foraging habitat of the Black-necked Crane reported biological threat as major. Biological threats as reported by communities include loss of habitat, food shortage and competition from other animals. We recommend the present roosting areas be designated as part of the conservation areas for Black-necked Crane wintering in Bumthang district. In addition to preserving these areas, government should also encourage farming in foraging habitats of Black-necked Crane, because they mainly feed on barley, wheat, paddy, potatoes and buckwheat, besides roots, tubers and insects in the wetlands.

## Background

Black-necked Crane (*Grus nigricollis)* is the only alpine crane species and the last of the world’s 15 crane species to be discovered by the Russian naturalist, Count Przhewalski near Lake Koko Nor in northeastern Tibet in 1876 (ICF 2012). The population of BNC is estimated at 10,070–10,970 individuals globally (Birdlife International 2012) and is classified as vulnerable under the IUCN Red List (IUCN 2009).

**Fig. 1:**
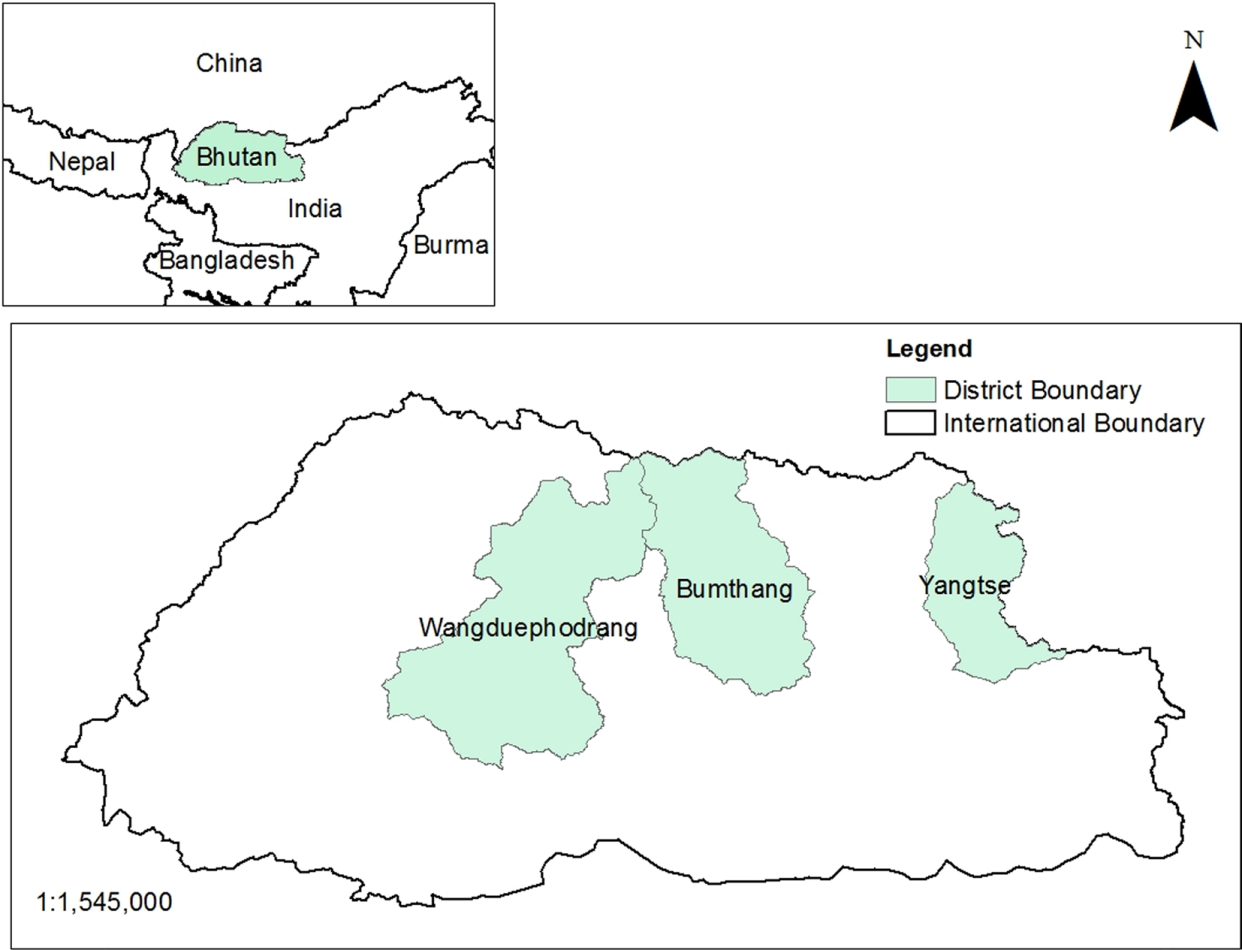
Map of Bhutan and districts with BNC winter roost

**Fig. 2:**
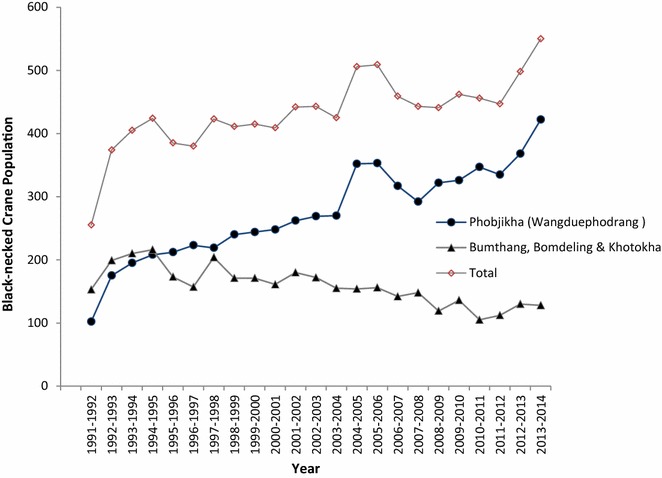
Population trend of BNCs visiting Bhutan

Bhutan is one of the major wintering sites for BNC, in addition to China and India. BNC reside in Bhutan from late October to mid-February (Lhuendup and Webb 2007) each year and the major winter habitats are in Phojikha and Khotokha in Wangduephodrang district, Bomdeling in Trashi Yangtse and Gyatsa in Bumthang districts (Fig. 1). The Population of BNC wintering in Bhutan is increasing (Fig. 2), and this is due to increasing number of BNC wintering in Phobjikha as per the record maintained by Royal Society for Protection of Nature (RSPN). However, record maintained by RSPN showed gradual decline in BNC visiting Bumthang, Bumdeling and Khotokha in Wangduephodrang district.

The roosting and foraging habitats of BNCs in Bumthang, Wangduephodrang and Trashi Yangtse have never been investigated and their habitat requirements are unknown. In this preliminary study, we identify current and former/abandoned BNC’s winter roosts, forage preferences and conservation threats based on local resident expertise and data available with government institutions and RSPN.

## Results and discussion

### Current roosting sites

**Fig. 3:**
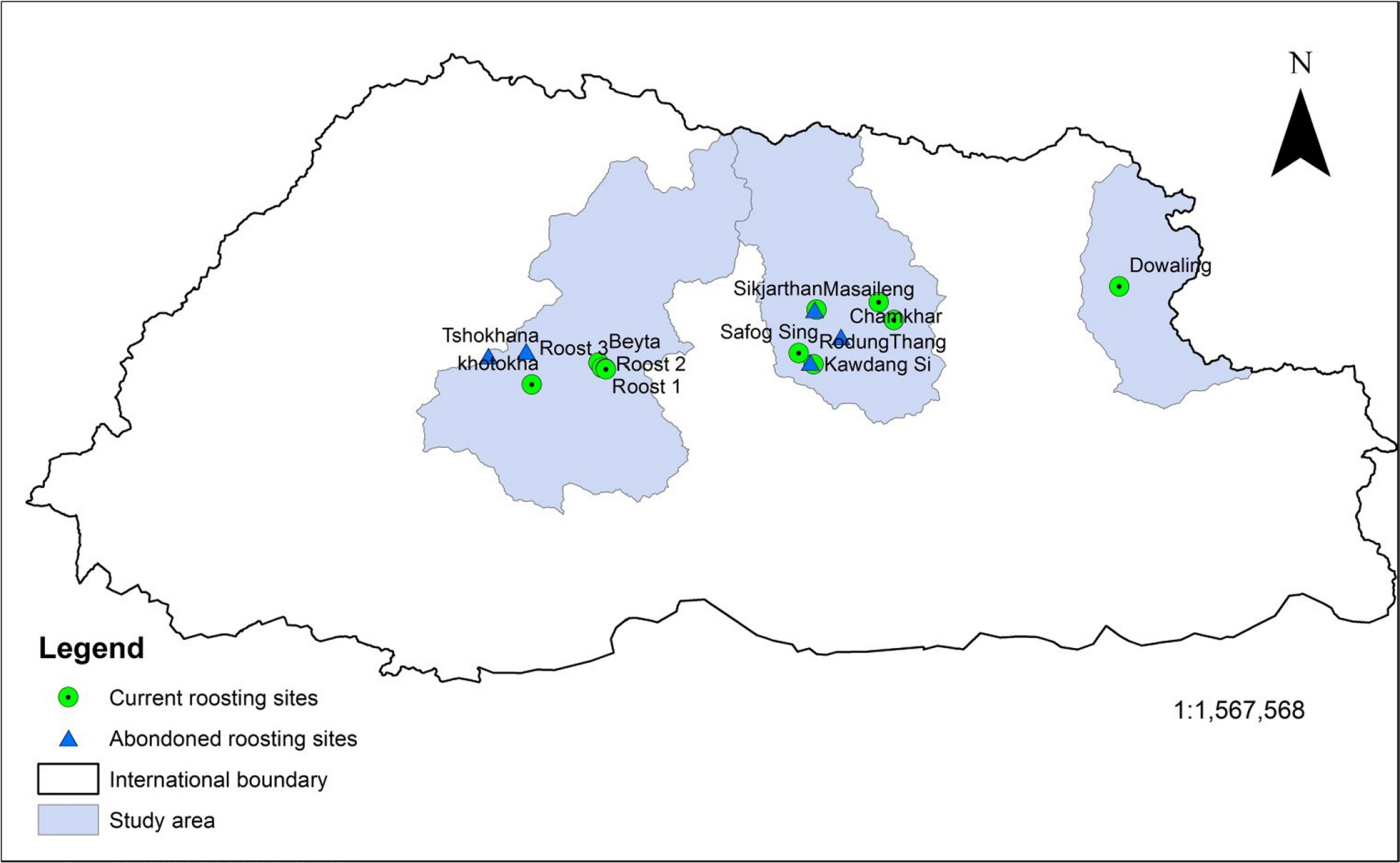
Current and abandoned roosting sites of cranes wintering in Bhutan

Five roosting habitats are currently used by BNCs in Bumthang district (two sites in Chhume and Tang Geog^1^ and one site in Chhokhor Geog). The roosting sites are Kawdang Singma (N27°29′01.0″, E090°41′10.9) and Sagfog Singma (N27°30′41.4″, E090°38′57.7″) in Chhume Geog. Tandigang (N27°38′12.5″, E090°50′47.1″) and Pralang (N27°35′33.7″, E090°53′03.5″) in Tang Geog and Sikjarthan (N27°37′04.3″, E090°41′35.5″) in Chhokhor Geog. Even in Wangduephodrang district, five roosting sites are used by cranes in the valley of Phobjikha and Khotokha. The roosting sites fall within the periphery of the main roost (N27°28′21.03″, E090°10′31.70″), roost-1 (N27°28′15.77″, E090°10′33.90″), roost-2 (N27°28′32.00″, E090°09′57.76″), Beyta (N27°29′16.35″, E090°09′28.12″) and Khotokha (N27°25′53.63″, E089°59′29.99″). Trashi Yangtse district has only one roosting site at Dowaling (N27°40′31.9″, E091°26′24.1″) in Bumdeling Geog (Fig. 3).

### Current foraging sites

The foraging habitats of the BNCs are mostly the agricultural fields, where barley, wheat and potatoes are cultivated besides the wetlands. In all the study sites, agriculture fields and wetlands in and around the settlements were foraging habitats. The BNCs forage within the radial distance of 5–10 km from their roosting sites.

### Abandoned roosting habitats

**Fig. 4:**
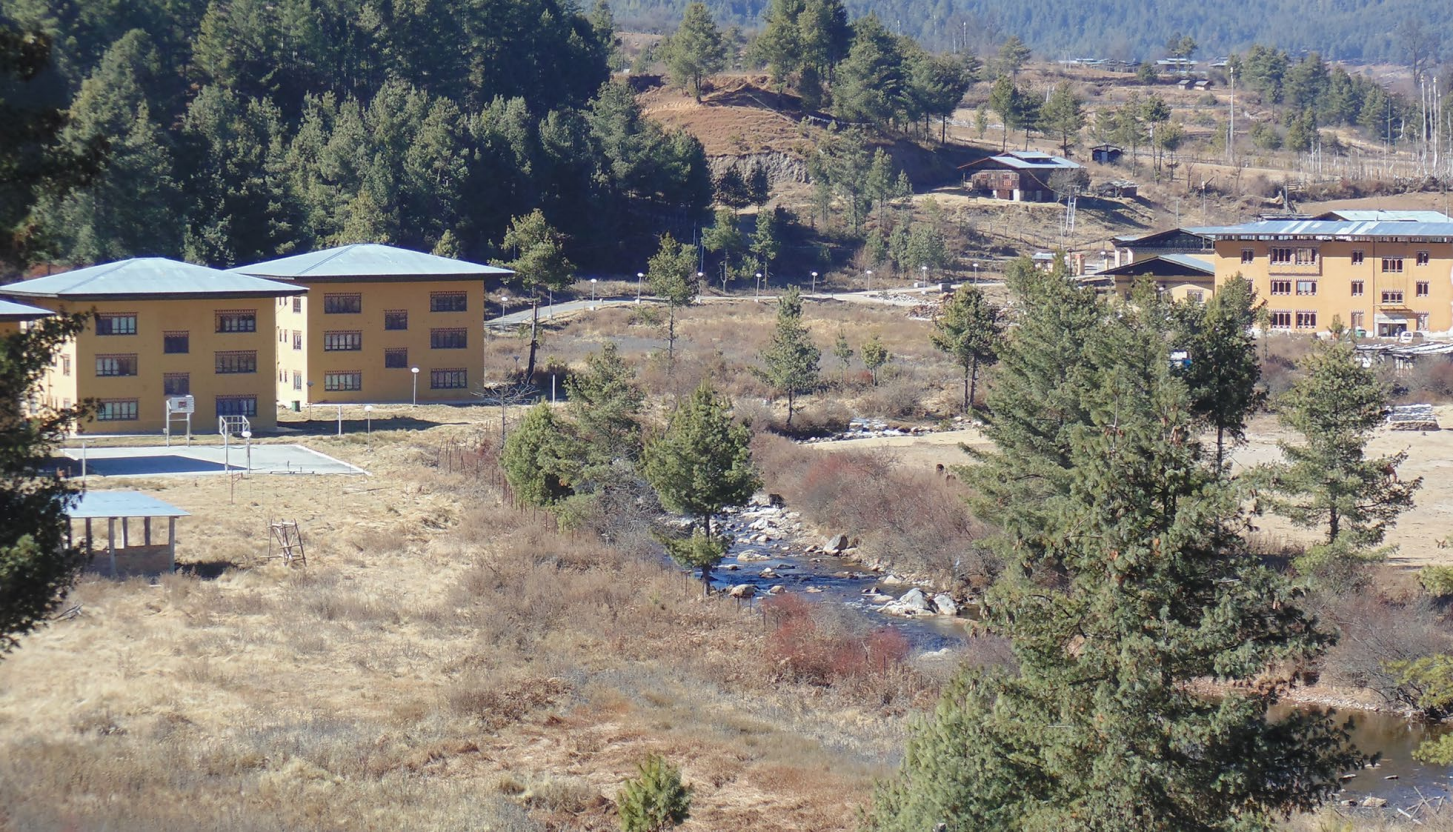
Rodhungthang—one of the former roosting habitats of BNC

The Black-necked Cranes have abandoned some roosting habitats in Wangduephodrang and Bumthang districts. In Wangduephodrang, the cranes abandoned roosting sites in Tshokhana and Samtengang, while in Bumthang they left roosting sites in Rodhungthang (Fig. 4), Masaileng and Chamkhar. The roosting habitats may have been abandoned due to the drainage of wetland for development of agricultural land and construction of infrastructure for the developmental activities. The foraging habitats may have been abandoned due to agriculture lands being left fallow by the people, resulting in the reduction of the food availability for cranes.

### Threats to Black-necked Cranes

The conservation threats to BNCs wintering in Bhutan as reported by the respondents are grouped into four major threats viz. biological, political, social and natural threats (Table 1). All threats, which has any link with the habitat and food availability are grouped as biological threats; those threats due to the changes in the policies and coming in of developmental activities are grouped as political threats; threats which are due to adverse anthropogenic factors such as trapping and hunting are grouped as social; and natural threats such as diseases, predators etc. are classed as natural threats.

**Table 1:** Conservation threats classification

Biological threats	Political threats	Social threats	Natural threats
Loss of habitats (roosting, foraging, feeding, etc.)	Conversion of wetland for other purposes	Hunting	Mortality
Food shortage	Lack of well defined law and policies in species conservation	Trapping	Predators
Competition		Killing	Diseases
Lack of scientific study		Disturbances	Environmental factors

Of the total 107 respondents, 47 % of the respondents were of the opinion that biological threat was the major conservation threat to BNCs in Bhutan, followed by social threat (18 %), natural threats (8 %) and political threat (7 %). However, 20 % of the respondents either said they do not know anything with regards to threats to BNC or preferred not to say anything.

Our questionnaire survey revealed that the trend of framing practice has decreased in the communities, as 68 % of the respondents reported of decrease in farming trend from a decade ago. We also found out that about 75 % of the respondents used to undertake agricultural practices in the foraging area of BNC about a decade ago. Though it is still practiced, but has decreased considerably. Our study found a strong correlation between the population of BNCs and the farming trend in the study area; r = .69, p < .05. This probably indicates that the number of BNCs visiting an area is dependent on the way people practice farming. Likewise biological threats is also significant on the decline in the BNC’s population, r = .73, p < .05. We found that, though the trend in agriculture practice has decreased the use of in-organic fertilizers like urea and NPK (Nitogen, Phosphorus and Potassium) has increased considerably. Only a decade ago, 78 % of the respondents didn’t use any kinds of in-organic fertilizers but now 89 % of the respondents admitted of using it. This may be having some relations to the habitat preference for BNC, but it warrants further research.

## Conclusions

The Black-necked Cranes have wintered in Bhutan since time immemorial. The oldest survey respondent (82 year old woman from Bumthang) stated “BNC has always visited our area since the time I could remember”. She also stated as BNCs being the part of Bumthang’s landscape and how people of the community welcomed the BNC whenever they visit the area with great happiness. However, people in Bumthang and Trashi Yangtse reported witnessing the decrease in BNC visiting their place each winter and the records maintained by RSPN corroborates the claim.

During the time of this study, the BNCs were sighted in all the three districts. The highest number of cranes (422) was sighted in Phobjikha roosting habitat in Wangduephodrang district, followed by Bomdeling in Trashi Yangtse district (112), Bumthang district (9) and 7 were sighted in Khotokha in Wangduephodrang district. Phobjikha in Wangduephodrang is a wide valley with open grasslands and dwarf bamboos. There is a stream meandering through the open grasslands, and farmlands occupy the peripheral slopes where potatoes and turnips are cultivated. Phobjikha has an area of about 162 km^2^, wherein about 5000 people (RSPN 2015) live along with their domestic animals and annual visitors, the BNC. Since good number of BNCs visit annually in Phobjikha, it warrants keeping domestic predators and anthropogenic disturbances at bay. We believe that adequate and frequent research should be undertaken to prevent possibility of the out-break of avian diseases.

Our study reflects the importance of local agriculture, as growing crops serves as good foraging sites for BNCs. Agricultural practices seems to have positive relations with the number of BNCs visiting the wintering sites in Bhutan, thus, it is of utmost importance for the government to initiate some form of mechanisms to encourage communities residing within the BNCs habitat to continue or encourage more farming activities. This may be achieved as about 91 % of the respondents believed that communities should be included in any conservation activities of BNCs.

Our study recognizes humans and their agricultural practices as an integral part of the BNCs existence. We would like to recommend all the areas, which are visited by BNCs to be brought under crane conservation area and to include communities in any conservation activities.

## Methods

The study was carried out in the wintering habitat of BNC in Bhutan (Fig. 1). The study covers three districts viz. Bumthang, Trashi Yangtse and Wangduephodrang. The field survey for habitat mapping was carried out using the Garmin *GPSmap 60CSx*. The coordinates of both former (based on local people’s information) and current roosting and foraging habitats within the study area were collected based on the information provided by the key informants. Key informants were identified based on the reference provided by the local people, who they believe would know more about BNC and its habitat within their locality. After mapping the roosting and foraging habitats with the help of key informants, a survey was conducted among a sample for those households falling within the 3 km radial distance of the roosting and foraging habitats to inquire about the perceived threats to BNC and to identify the change in land use pattern, if any. A total of 107 households were interviewed; 8 villages with 32 households from Trashi Yangtse district, 7 villages with 53 households from Wangduephodrang district and 5 villages with 22 households from Bumthang district. The sample size for questionnaire survey for each districts were determined using Yamane formula (Yamane 1967). For the questionnaire survey, efforts were made to interview head of households and old people (above 60 years of age) with the assumption that they know more about its habitats. In absence of the head of households, oldest members present in the family, at the time of the visit, were interviewed. However, efforts were also made to involve both the gender equally for the interview.

The focused group discussions in the form of public meetings were also conducted in all the study areas to gather additional information on the conservation threats and the location of the habitats. The data gathered were further crosschecked through Gups,^2^ village Tshogpas,^3^ Renewable Natural Resources (RNR) extension staff, local forest officials and non-governmental organizations.

## Abbreviations

BNC: Black-necked Crane
RSPN: Royal Society for Protection of Nature
RNR: Renewable Natural Resources

## Footnotes

1 Administrative Block.

2 Elected head of an administrative block.

3 Elected head of a village.
